# Remarks on the Treatment of Gleet by Local Remedies

**Published:** 1861-07

**Authors:** William C. Otterson

**Affiliations:** Brooklyn, New York


### ART. III.—*Remarks on the Treatment of Gleet by Local Remedies.*

By WILLIAM C. OTTERSON, M.D., of Brooklyn, New York.

THE slight discharge from the urethra so often met with after an attack of gonorrhœa, and known as chronic blenorrhagia, gleet, or chronic gonorrhœa, occurs under various circumstances, and its seat, cause, and treatment has been a source of much diversity of sentiment among authors. Cooper, Vidal, Acton, Hunter, Chelius, and others differ in their views in regard to the seat and pathology of the disease, yet they all concur in the general plan of treatment, namely, stimulating and astringent injections, cubebs, and terebinthinates, and speak, as a last resort, of the use of the bougie.

We find these writers placing the seat of the disease in the prostate, Cowper's glands, the lining membrane of the urethra, the lacunæ, and the navicular fossa. I am disposed to think that Sir A. Cooper was nearest right, and that the glans penis and the lacunæ therein are the real seat of this affection. What the precise pathological condition of the glans is, I am unable to say, but it would appear that, owing to its delicate structure and the fact of its being the most frequent seat of acute attacks, the onus of the chronic form of the disease should be centred upon this portion of the organ; and that many of the most obstinate cases of gleet, in the absence of stricture and disease of the prostate, are dependent upon a morbid condition of the glans, and not upon any disease or change of the lining membrane of the urethra itself. My reasons for this belief are: 1. The amount and character of the discharge. 2. The point of irritability upon passing an instrument. 3. The inadequacy of injections to control the disease permanently. 4. The failure of internal medication or hygienic measures to exert any special influence over it.

1. The discharge is usually found to be nothing more than opaque mucus, varying in consistence according to circumstances. This in the morning occupies the meatus, at times almost blocking it up, and is easily removed if the patient runs the finger along the under surface of the urethra, beginning about an inch from the meatus, when the secretion escapes. After that the urethra may be squeezed from the prostatic region to the meatus, and yet no more of the discharge be procured. 2. In passing an instrument, when about an inch from the meatus, and corresponding with the navicular fossa, the patient complains of pain; and after the instrument passes this point the whole tract of the urethra beyond is discovered to be free from irritability. This I have found as an almost invariable result in my cases of obstinate and long-standing gleet when there was no stricture. 3. Injections of all kinds have utterly failed to make any permanent impression upon the discharge, though there have been remissions for a few days at different times. 4. Internal remedies have failed to exert any perceptible influence over it.

If the lining membrane of the urethra alone were involved, the result of medication would be different, for it is well known that, in acute gonorrhœa, many of what are called specific medicines do exert a modifying and decidedly beneficial influence over the diseased tract of the canal. But in this form of gleet they fail, because their action is not adapted to the disease of this particular structure. If Cowper's glands are the seat, then the application which I make in these cases would appear to be useless, as the remedies are not applied to the portion of the canal where they are situated.

As preliminary to the treatment I first examine the patient to ascertain if there is stricture or narrowing of the canal, and if I find this to be the case, the usual measures are adopted to overcome it. Sometimes this alone will effect a cure; but if the discharge continues, I introduce a No. 8 metallic grooved bougie, which is applicable to most cases; the sizes may vary from No. 6 to No. 10, the groove being filled with citrine ointment or calomel: the latter I prefer as being more certain in its action. The instrument need be passed only a sufficient length up the urethra to get the point beyond the glans penis, when it should be carefully revolved several times. It should be allowed to remain in for three, four, or five minutes; and then a probe, adapted to the curve of the instrument, should be passed down the groove; if they are then withdrawn simultaneously the whole of the calomel or ointment will be left in apposition with the parts. This may be repeated twice or thrice a week, at the same time that the bowels are kept soluble. Upon withdrawing the instrument a little smarting may be complained of, and perhaps a few drops of blood issue, but this is not important. If this plan of treatment is followed assiduously, the patient may be assured with much confidence that a very short time only will be required to remove every trace of the most obstinate and hitherto intractable gleet.

Within the last few years, I have treated successfully cases of acute gonorrhœa, spermatorrhœa, and irritable urethra with little modification of the practice as here laid down.

I look upon this plan as preferable to any other, as it is less painful to the patient. After a few introductions of the instrument, the urethra becomes accustomed to its presence, and no inconvenience is experienced.

I have had quite a large number of these cases under my care, and having formerly been in the habit of adopting the old stereotyped practice, have seen many of them linger from month to month, and finally pass into other hands. Since, however, I have become convinced that *benefit can be derived only from direct alterative medication to the seat of the disease*, and have pursued the plan of treatment laid down in this paper, I have not failed to cure a single case, marked benefit arising after a few applications, even under apparently the most unfavorable circumstances.

As some authors have recommended the use of mercury, with a view to produce its specific effect upon the system, I transfer one case from my note-book, illustrating its action in this affection.

A man consulted me on account of a chancre, which I cauterized with solid nitrate of silver, and after giving a cathartic kept him on blue mass with opium, until its specific action became manifest, and as there was some induration, kept up a slight mercurial action for some time by iodide of potassium and compound extract of sarsaparilla. This treatment being continued about two weeks the chancre healed kindly. The patient now, for the first time, informed me that he had been suffering from gleet for eight months, which, at the time he first applied, was quite severe; but thinking that the medication and regimen adopted for the cure of the syphilis would also suffice for his gleet, he did not mention it till after his primary disease was cured; but finding he was not free from his old discharge he wished further advice. I proposed the bougie, but having been present when I had introduced it on his friend, he desired that some other measure should first be tried. I consented, after giving an unfavorable prognosis. The old routine of injections was gone through with, and after one month's trial he determined to submit to the medicated bougie. Upon examination, no irritable point was found after the end of the instrument had passed the navicular fossa, nor was there any stricture or narrowing of the urethra. A perfect cure was effected in about three weeks, no relapse following.

A gentleman applied to me on account of a gleet, the sequel of a gonorrhœa, which had troubled him about five months, and for which he had treated himself with injections and balsam of copaiba.

Upon examination, I found some narrowing of the canal just anterior to the bulb, with irritability at this point and at the navicular fossa. This constriction readily gave way to the use of graduated bougies, but the gleet continued. I treated him upon the old plan for six months; all the different remedies were tried, and strict rules of regimen enjoined. During this period there were several remissions, but nothing permanent. At last I introduced the medicated bougie twice a week, at the same time removing all dietetical restrictions. At the end of six weeks, although he daily drank large quantities of beer, he was entirely cured

Some may be disposed to think that this case required tonics and nourishing diet, but these had been given as one of the changes during the six months of the old treatment.

In spermatorrhœa and irritable urethra the adjuvanta necessary to a cure will readily suggest themselves, both hygienic and therapeutic, such as cold bathing, friction, and proper ventilation in the sleeping apartment, with, perhaps, the internal use of iron, nux vomica, and quinine.

